# Adequacy of Cancer Screening in Adult Women with Congenital Heart Disease

**DOI:** 10.1155/2013/827696

**Published:** 2013-08-01

**Authors:** Mitalee P. Christman, Margarita Castro-Zarraga, Doreen DeFaria Yeh, Richard R. Liberthson, Ami B. Bhatt

**Affiliations:** ^1^Massachusetts General Hospital, Harvard Medical School, Boston, MA 02114, USA; ^2^University of Massachusetts, Worcester, MA 01655, USA

## Abstract

Adults with congenital heart disease (ACHD) face noncardiac healthcare challenges as the population ages. We assessed whether women with ACHD have comparable cancer screening rates to non-ACHD women in a cardiac practice and to the general population. We performed a retrospective review of 175 adult women seen in a cardiac care center in 2009–2011. Data on Pap tests, mammography, and colonoscopies, were collected through electronic medical records and primary care provider records. Adequate documentation was available for 100 individuals with ACHD and 40 comparator cardiac patients. The adequacy of screening was determined using guidelines set forth by the American Cancer Society in 2010. Compared with the national average, ACHD patients had significantly lower rates of Pap tests (60% versus 83%, *P* < 0.001) and mammography (48% versus 72%, *P* < 0.001). Compared with non-ACHD women in the same practice, ACHD patients had consistently lower rates of mammography (48% versus 81%, *P* = 0.02) and colonoscopies (54% versus 82%, *P* = 0.23). As the population of ACHD individuals ages, attention to cancer screening becomes increasingly important but may be overlooked in this population. Primary care physicians and cardiologists should collaborate to ensure appropriate cancer screening for this growing population.

## 1. Background

As a result of surgical advances as well as improvements in diagnosis and medical management, there may now be over 1 million adults with congenital heart disease in the United States, and this population is rapidly expanding [1]. As this population ages, the annual incidence of age-related malignancy rises, and caregivers must now ensure adequate and timely preventive cancer screening for the adult congenital heart disease (ACHD) patient.

Primary care of the ACHD patient may be overshadowed by late cardiac complications including arrhythmia, valvular disease, and heart failure [2, 3]. The European Society of Cardiology and 2008 American College of Cardiology/American Heart Association guidelines have both called for increased access to primary care physicians and attention to health maintenance [4, 5].

To develop strategies to improve overall screening, it is essential to identify groups of individuals with inadequate screening rates and to assess potential barriers to preventive screening. Known risk factors for poor participation in preventive screening include low socioeconomic status, lower levels of education, immigrant status, lack of health insurance and importantly, lack of a primary care provider [6]. Individuals with chronic disease rely on subspecialists for their medical care and may not have a primary care physician or gynecologist thus reducing the likelihood of access to general preventive care [7, 8]. Even among chronic disease patients who regularly follow up with primary care providers there appears to be an overall reduction in participation with preventive care [9, 10]. ACHD patients may have additional unique barriers to adequate cancer screening. They are frequently lost to followup during adolescence and early adulthood, for several reasons, including inadequate understanding by patients and parents of the importance of life-long medical care [11] and challenges to transition from adolescent to adult care [12, 13].

In this study, we investigate the documentation of preventive cancer screening provided to women with congenital heart disease. We hope to raise awareness among cardiologists and primary care physicians alike regarding the essential need for preventive screening for treatable diseases in this growing and aging ACHD patient population.

## 2. Methods

This retrospective institutional review board approved study of women over 18 years of age was performed at a single ACHD center. Primary care for these individuals was provided by several primary care practices including community centers, multispecialty practices, and academic centers. One hundred seventy five patients with ACHD seen in the cardiology practice were consecutively selected between 2009 and 2011. Individuals with isolated patent foramen ovale, mitral valve prolapse, or isolated bicuspid aortic valve were excluded from the ACHD cohort. One hundred ACHD patients had adequate medical record data for analysis. Data regarding medical history, sexual history, pap smears, colonoscopy, and mammography were obtained from patient medical records, either from the electronic medical record or from primary care physicians' faxed documentation.

Adequacy of screening was determined using criteria set forth by the American Cancer Society (ACS) in 2010 (Table 1) [14]. While there is consensus among organizations regarding the intervals for cervical screening [14, 15] and colonoscopy [14, 16], there has been controversy regarding the recommended mammography interval. Although the US Preventive Services Task Force guidelines call for mammography every 2 years after age 50 [17], many groups including ACS, American College of Gynecologists, and American College of Radiology/Society of Breast Imaging continue to recommend annual mammography after age 40 years [14, 18, 19], the criteria which were applied in our current analysis.

**Figure 1:**
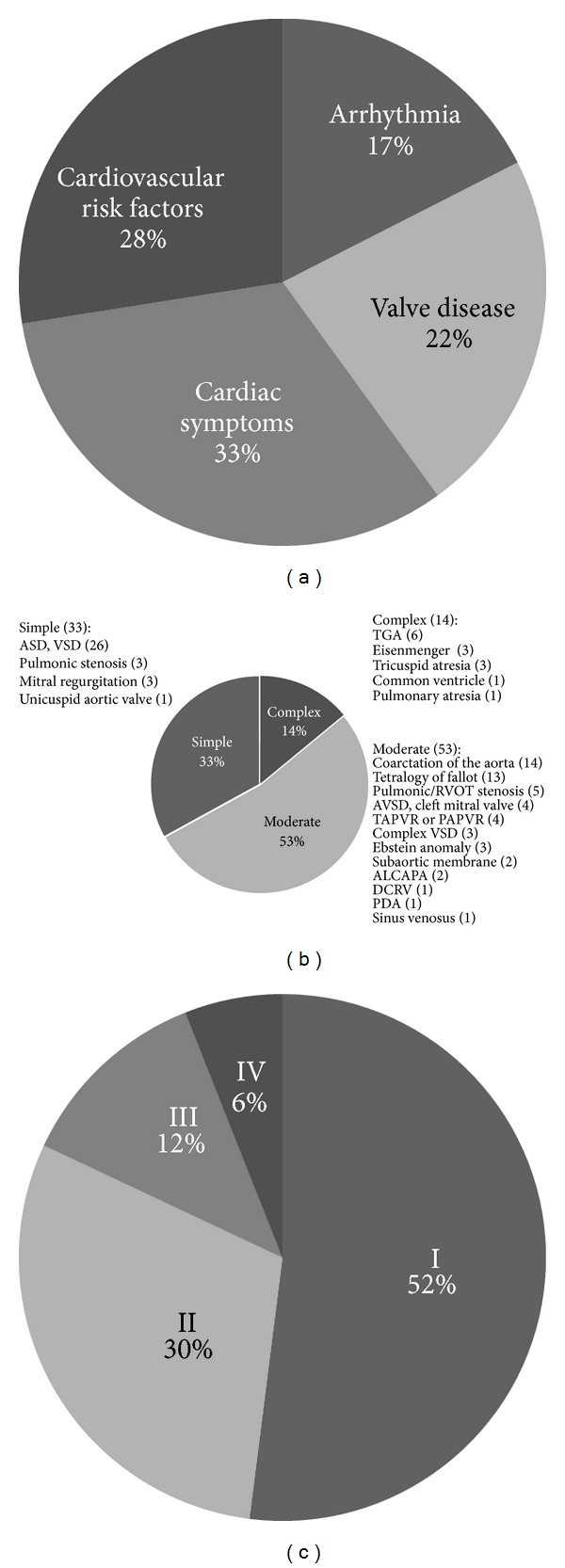
(a) The control group contained 40 women without congenital heart disease who presented with cardiac symptoms such as chest pain or palpitations, cardiovascular risk factors, valvular disease, or arrhythmia. (b) The study group contained 100 women with congenital heart disease whose lesions spanned the spectrum of the Bethesda classification [20] with simple, moderate, and complex lesions all represented. (c) Using the New York Heart Association Functional Classification, most women in the study group had either no limitation in ordinary physical activity (Class I) or mild symptoms (Class II). Fewer subjects had marked limitation in activity due to symptoms (Class III) or symptoms at rest (Class IV).

Forty individuals with simple noncongenital cardiac disease (Figure 1(a)) seen in the same practice and age matched to within 3 years were consecutively selected as a comparator population. The congenital heart disease study group's screening rates were also compared to the Healthy People 2010 targets [21], ten-year national objectives established by the US Department of Health and Human Services to be achieved by 2010, contemporary to this study, and to US population screening rates determined by the Centers for Disease Control and Prevention in 2010 [22].

Statistical analysis was performed using EpiCalc 2000 (Brixton Health) [23]. Continuous variables were assessed using the student's *t*-test. Categorical variables were assessed with the Chi-square test. Significance was measured at a level of *P* < 0.05.

## 3. Results

### 3.1. Baseline Demographics

Individuals with ACHD had a median age of 38 years (SD 14) (Table 2), with 33% simple, 53% moderate, 14% complex disease severity per the Bethesda classification [20] (Figure 1(b)). They demonstrated variable severity based on New York Heart Association (NYHA) class (Figure 1(c)). The majority of individuals identified their race as Caucasian. There was a broad range of congenital heart lesions encompassed by this population (Figure 1(b)).

### 3.2. Breast Cancer Screening

**Figure 2:**
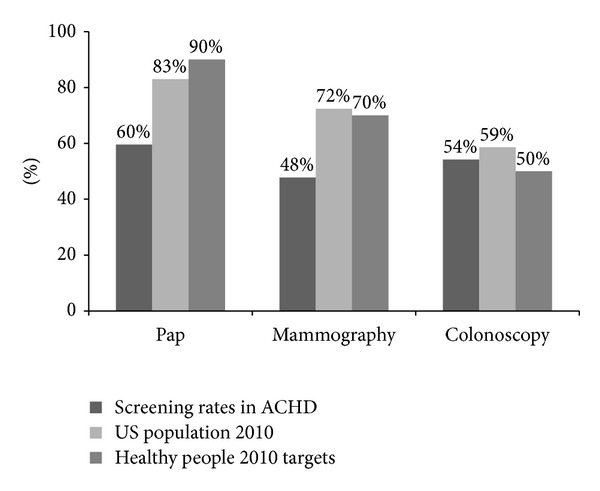
These bar graphs compare the study group's screening rates for cervical cancer (*n* = 94), breast cancer (*n* = 44), and colon cancer (*n* = 24) with those of the US population [22] and the Healthy People 2010 target screening rates [21].

Breast cancer screening rates were significantly lower in the ACHD population when compared with the US national screening rate as well as the local comparator population (Table 3; Figure 2). Mammography was performed in only 48% of the ACHD group compared to 81% of the non-ACHD cardiac group (*P* = 0.02) and 72% of the US population in 2010 (*P* < 0.001). The ACHD group fell short of the national mammography target of 70%.

### 3.3. Cervical Cancer Screening

Adequate cervical cancer screening was seen in 60% of both ACHD and comparator groups, compared with 83% of the US population in 2010 (*P* < 0.001). The national target for cervical cancer screening was 90%, with all three populations falling short of the target screening rate.

### 3.4. Colon Cancer Screening

Adequate colon cancer screening was performed in 54% of the ACHD population versus 81% of comparator group (*P* = 0.23), with a national screening rate of 58% (*P*-value for ACHD versus national population 0.82). The national target for colon cancer screening was 50%, with all three populations achieving the target screening rate.

### 3.5. ACHD Disease Severity and Screening Rates

Individuals with simple, moderate, and complex disease had 58%, 37%, and 44% adequate overall screening, respectively. Adequacy of screening decreased as cardiac symptoms increased based on NYHA class (50% NYHA Class I, 47% NYHA Class II, 25% NYHA Class III, 17% NYHA Class IV; *P* = 0.2) (Table 4).

### 3.6. Screening Results and Followup

All ACHD and cardiac control group subjects had a primary care physician. There was documentation of followup for all abnormal results in the study population (Table 5).

## 4. Discussion

The ACHD population continues to increase in the US, and both adult and pediatric cardiology practices as well as primary care providers are seeing an influx of these individuals. As the population ages, the incidence of cancer will rise in this population as well. Our study is the first to examine the rates of cancer screening documented among ACHD patients.

The overall screening rate for breast, cervical, and colon cancer was 45% in the ACHD population, with a failure to achieve target screening goals or meet the national screening averages in breast cancer and cervical cancer screening. Colon cancer screening rates for the ACHD population were similar to the national average and surpassed the Healthy People 2010 target. Colonoscopy rates in the local cardiac patient control group were higher, though not statistically significant. This may be due to the control group's composition of overall healthier individuals with arrhythmia, mild valvular disease, fewer cardiac symptoms, and cardiovascular risk factors, reflecting a proactive patient population.

Barriers to adequate cancer screening in this population are likely multifactorial and include both patient and physician factors. Patients with chronic disease and survivors of potentially life-threatening conditions may place less value on screening and preventive treatment [10, 24] possibly contributing to lower cancer screening rates. The significant discrepancy in breast cancer screening may stem from ACHD patient hesitancy to undergo mammography. Perceived procedural discomfort in individuals with a prior sternotomy, potential additional radiation exposure after repeated cardiac imaging, and body image issues have all been anecdotally cited. If ACHD survivors rely primarily on cardiologists for routine medical care, they may fail to receive cancer screening services.

Individuals with worse functional status (higher NYHA Class) had lower rates of cancer screening. As the severity of an individual's disease increases, competing medical comorbidities, patient hesitation to undergo additional testing, or physician choice to forego testing in patients with limited life expectancy may influence screening compliance rates.

## 5. Study Limitations

The limitations of this study are those common to retrospective medical record based analyses including a dependence on clinical detail provided and a lack of information about patient preferences or provider decision making. Socioeconomic characteristics were not robust in this database. The overall rate of cervical cancer screening is low in both groups, and it is possible that some patients may have a local gynecologist, and those Pap tests may not have been captured in our database. Additionally, our analysis is limited because the overall number of patients eligible for colonoscopy is low. Conversely, the individuals studied were drawn from a population that attended cardiology clinic and have access to primary care. This may lead to overestimation of screening compliance rates, as many ACHD individuals nationwide are lost to followup and do not have primary care physicians and are therefore less likely to undergo cancer screening.

## 6. Conclusions

As the population of individuals with ACHD continues to grow and age, screening for preventable disease becomes an important responsibility of their care providers. The national targets for cancer screening rates continue to rise. The Healthy People 2020 target for breast cancer screening is 81%, cervical cancer screening is 93%, and colon cancer screening is 70% [25]. To meet these goals, cardiac providers and primary care physicians must define their roles in long-term management of ACHD patients. Patient education regarding the importance of health maintenance and cancer screening should be a priority for providers and patient advocacy groups.

Over the past 40 years, each decade has brought advances in medical care and quality of life to adults with congenital heart disease. ACHD caregivers and patients have redirected conversations from exercise restriction to exercise encouragement and have recognized that contraception and family planning are essential discussions, and we now, as a community, face the challenge of addressing noncardiac issues associated with increased life expectancy. Promoting cancer screening may not only save lives, it is the next opportunity to transform our approach to the ACHD population's overall care.

## Figures and Tables

**Table 1:** Guidelines and criteria for determining cancer screening adequacy.

	Age of eligibility	Screening adequate if	
		No. tests obtained	Within (yrs)*
Pap	21	2	*T* − 3
Mammography	40	2	*T* − 2
Colonoscopy	50	1	*T* − 10

*T is the year of enrolment or age 70, 75, and 75 for Pap tests, mammograms, and colonoscopies, respectively. Screening was deemed adequate if at least no. tests were obtained in a specified time interval prior to enrolment [14].

**Table 2:** Baseline Demographics.

	ACHD (*n* = 100)	Control (*n* = 40)	Total
Median age in years (SD)	38 (14)	42 (15)	*P* value 0.2 NS
18–40	59	19	78
41–50	20	11	31
51–64	14	6	20
>65	7	4	11
Race			
White	89	33	122
African American	1	3	4
Asian	4	2	6
American Indian	0	0	0
Native Hawaiian/Pacific Islander	0	0	0
Hispanic	6	1	7
More than one race or other	0	0	0
No data	0	1	1

**Table 3:** Screening rates in adult congenital heart disease group versus control group versus 2010 US population screening rates.

Test	ACHD (*n* = 100)	ACHD	Control (*n* = 40)	Control	*P* value	US Pop	US Pop	*P value
	No. eligible	Screening rate	No. eligible	Screening rate		No. eligible	screening rate [22]	
Pap	94	60%	35	60%	0.87	8999	83%	**<0.001**
Mammogram	44	48%	21	81%	**0.02**	4869	72%	**<0.001**
Colonoscopy	24	54%	11	82%	0.23	8914	59%	0.82

*P value for ACHD compared with US population screening rate.

**Table 4:** Screening rates by New York Heart Association class.

	No. ACHD patients in class (*n* = 100)	% adequately screened (*P* = 0.22)
I	52	50%
II	30	47%
III	12	25%
IV	6	17%

**Table 5:** Followup of abnormal results.

	ACHD (*n* = 100)		Control (*n* = 40)	
	No. abnormal	Rate of followup	No. abnormal	Rate of followup
Paps	12	83.33%	2	100%
Mammograms	3	100%	3	100%
Colonoscopies	10	70%	3	100%
